# Supplementary material for: Effects of acupuncture on age-related macular degeneration: A systematic review and meta-analysis of randomized controlled trials
Source: PLoS One. 2023 Mar 23;18(3):e0283375. doi: 10.1371/journal.pone.0283375 (PMC10035922; doi:10.1371/journal.pone.0283375)
Supplement: S1 Appendix — (PDF) [file pone.0283375.s002.pdf]

Supplement 1 Search strategy

Table 1 Search strategy

No	Database (Number of articles retrieved)	Search items
1	PUBMED (n = 3)	1.("Randomized Controlled Trial" [Publication Type]) OR ("Randomized Trial" [All Fields]).<br>2. ("acupuncture"). [MeSH Terms]<br>3. ("exp acupuncture therapy" OR "acupuncture" OR "acupressure" OR "acupoint" OR "electroacupuncture" OR "electro-acupuncture" OR "elongated needle" OR "warming needle" OR "auricular acupuncture"). [All Fields]<br>4. or/2,3.<br>5.(Age-Related Macular Degeneration[MeSH Terms])<br>6.("Degeneration, Macular" OR "Macular Degenerations" OR "Maculopathy" OR "Maculopathies" OR "Macular Dystrophy" OR "Dystrophy, Macular" OR "Macular Dystrophies" OR "Age-Related Macular Degeneration" OR "Age Related Macular Degeneration" OR "Age-Related Macular Degenerations" OR "Macular Degeneration, Age-Related" OR "Macular Degeneration, Age Related" OR "Maculopathies, Age-Related" OR "Maculopathy, Age-Related" OR "Maculopathy, Age Related" OR "Age-Related Maculopathies" OR "Age Related Maculopathies" OR "Age-Related Maculopathy" OR "Age Related Maculopathy"). [All Fields]<br>7. or/5,6.<br>8."humans". [MeSH Terms]<br>9. 1 and 4 and 7 and 8.
2	Cochrane (n = 4)	("Acupuncture") AND ("Randomized Trial") AND ("Randomized Controlled Trial") AND ("Age-Related Macular Degeneration" OR "Degeneration, Macular" OR "Macular Degenerations" OR "Maculopathy" OR "Maculopathies" OR "Macular Dystrophy" OR "Dystrophy, Macular" OR "Macular Dystrophies" OR "Age-Related Macular Degeneration" OR "Age Related Macular Degeneration" OR "Age-Related Macular Degenerations" OR "Macular Degeneration, Age-Related" OR "Macular Degeneration, Age Related" OR "Maculopathies, Age-Related" OR "Maculopathy, Age-Related" OR "Maculopathy, Age Related" OR "Age-Related Maculopathies" OR "Age Related Maculopathies" OR "Age-Related Maculopathy" OR "Age Related Maculopathy")

3	MEDLINE<br>(n = 2)	1. randomized controlled trial.pt.<br>2. controlled clinical trial.pt.<br>3. randomized.ab.<br>4. randomly.ab.<br>5. placebo.ab.<br>6. trial.ab.<br>7. groups.ab.<br>8. or/1–7.<br>9. exp animals/ not humans. sh.<br>10. 8 not 9.<br>11. ("acupuncture").ab.<br>12. ("Age-Related Macular Degeneration" OR "Degeneration, Macular" OR "Macular Degenerations" OR "Maculopathy" OR "Maculopathies" OR "Macular Dystrophy" OR "Dystrophy, Macular" OR "Macular Dystrophies" OR "Age-Related Macular Degeneration" OR "Age Related Macular Degeneration" OR "Age-Related Macular Degenerations" OR "Macular Degeneration, Age-Related" OR "Macular Degeneration, Age Related" OR "Maculopathies, Age-Related" OR "Maculopathy, Age-Related" OR "Maculopathy, Age Related" OR "Age-Related Maculopathies" OR "Age Related Maculopathies" OR "Age-Related Maculopathy" OR "Age Related Maculopathy").ab.<br>13. 10 and 11 and 12.
4	Embase<br>(n =8)	(Age-Related Macular Degeneration, acupuncture, randomized controlled trial, randomized) using a combination of multi-fieldsearch in all fields and EMTREE
5	China National Knowledge Infrastructure<br>(n = 58)	("Acupuncture" OR "electroacupuncture" OR "warm needle") [MESH] AND ("Age-Related Macular Degeneration" OR "Macular Degenerations") [MESH]
6	SINOMED<br>(n = 66)	1.("Acupuncture" OR "electroacupuncture" OR "warm needle"). [Common field]<br>2. ("Age-Related Macular Degeneration" OR "Macular Degenerations"). [Common field]

		3. 1 and 2.
7	Wanfang<br>(n = 54)	1.("Acupuncture" OR "electroacupuncture" OR "warm needle"). [Common field]<br>2. ("Age-Related Macular Degeneration" OR "Macular Degenerations"). [Common field]<br>3. 1 and 2.
8	Technology Periodical Database (VIP)<br>(n = 30)	1.("Acupuncture" OR "electroacupuncture" OR "warm needle"). [MESH or keywords]<br>2.("Age-Related Macular Degeneration" OR "Macular Degenerations"). [MESH or keywords]<br>3.1 and 2.
9	www.clinicaltrials.gov<br>(n = 1)	("Acupuncture" AND "Age-Related Macular Degeneration") AND ("randomized controlled trial" OR"randomized trial")
10	www.clinicaltrialsregister.eu<br>(n = 0)	("Acupuncture" AND "Age-Related Macular Degeneration") AND ("randomized controlled trial" OR"randomized trial")
11	trialsearch.who.int<br>(n = 0)	("Acupuncture" AND "Age-Related Macular Degeneration") AND ("randomized controlled trial" OR"randomized trial")
Mainly excluded literature (with reasons)		[1] Zhu JL, Gu ZY, Liu YL, et al. Clinical observation of acupuncture in the treatment of dry macular degeneration [J]. Shanghai Journal of Acupuncture and Moxibustion, 2018;37(06): 630-634. (comparison of different acupoints)<br>[2] Li G, Shao Y, Yin J. Early age-related macular degeneration treated with emayaoling acupuncture technique: a randomized controlled trial. Zhongguo Zhen Jiu. 2017;37(12):1294-1298. (comparison of different acupuncture techniques)<br>[3] Zhang X, Wang WY. Clinical study of Huatan Sanyu Mingmu Decoction combined with acupuncture in the treatment of exudative age-related macular degeneration [J]. Electronic Journal of Clinical Medical Literature, 2020;7(44):157+160. (inappropriate comparison)<br>[4] Zhu HY, Zou JS. Analysis on therapeutic effect of Professor Zou Jusheng’s emolliating liver and fortifying spleen method combined with acupuncture treating age-related macular degeneration[J].China Journal of Chinese Ophthalmology, 2015;25(04):265-268. (inappropriate comparison)

	[5] Lu AH. Clinical study of Huangbanfuming decoction combined with acupuncture in the treatment of age-related macular degeneration [D]. Hebei Medical University, 2016. (inappropriate comparison)<br>[6] Li T. Efficacy of Huangban Fuming Decoction combined with acupuncture in the treatment of age-related macular degeneration and its effects on macular edema, subjective symptoms, serum VEGF, PDGF and ES levels [J]. Journal of Sichuan of Traditional Chinese Medicine, 2018;36(12):174-177. (inappropriate comparison)

Supplement 2 Evaluation criteria of curative effect based on the “Criteria of Diagnosis and Therapeutic Effect of Internal Diseases and Syndromes in Traditional Chinese Medicine”

File 1 Evaluation criteria

1) Valid, with at least one of the following manifestations: visual acuity improved by 1 line or more; improvement of macular lesions in the fundus (exudation, partial absorption of hemorrhage); improved visual field;

2) Invalid: no change or progressive decrease in visual acuity; no change or aggravation of macular exudation and hemorrhage; no change or aggravation of visual field.

Supplement 3 Summary of findings

Table 2 Grading of evidence

Outcomes	Risk of bias	Inconsistency	Indirectness	Imprecision	Other considerations	Certainty of the evidence
**Clinical effificacy rates**	Serious[a]	Not serious	Not serious	Serious[b]	None	⊕⊕◯◯ LOW
Acupuncture alone	Serious[a]	Not serious	Not serious	Serious[bc]	None	⊕◯◯◯ VERY LOW
Acupuncture as an adjunct	Serious[a]	Not serious	Not serious	Serious[bc]	None	⊕◯◯◯ VERY LOW
**BCVA**	Serious[a]	Very serious[d]	Not serious	Very serious[be]	None	⊕◯◯◯ VERY LOW
**CMT**	Serious[a]	Serious[f]	Not serious	Very serious[be]	None	⊕◯◯◯ VERY LOW

GRADE Working Group grades of evidence

High certainty: We are very confident that the true effect lies close to that of the estimate of the effect

Moderate certainty: We are moderately confident in the effect estimate: The true effect is likely to be close to the estimate of the effect, but there is a possibility that it is substantially different

Low certainty: Our confidence in the effect estimate is limited: The true effect may be substantially different from the estimate of the effect

Very low certainty: We have very little confidence in the effect estimate: The true effect is likely to be substantially different from the estimate of effect

Explanations

a. Concerns about bias in the domains of allocation concealment, blinding of outcome assessment and selective reporting.

b. Small sample studies accounted for the majority.

c. Concerned about the number of studies and participants.

d. I-squared= 89%.

e. Wide confidence intervals around estimate.

f. I-squared= 67%
